# Defining competence profiles of different medical specialties with the requirement-tracking questionnaire – a pilot study to provide a framework for medial students’ choice of postgraduate training

**DOI:** 10.1186/s12909-020-02479-6

**Published:** 2021-01-12

**Authors:** Elena Zelesniack, Viktor Oubaid, Sigrid Harendza

**Affiliations:** 1 III. Department of Internal Medicine, University Medical Center Hamburg-Eppendorf, Martinistr. 52, D-20246 Hamburg, Germany; 2 German Aerospace Center (DLR), Hamburg, Germany

**Keywords:** Competence, Medical specialties, Requirement analysis, Postgraduate training

## Abstract

**Background:**

The medical specialties are characterised by a great diversity in their daily work which requires different sets of competences. A requirement analysis would help to establish competence profiles of the different medical specialities. The aim of this pilot study was to define competence profiles for individual medical specialties. This could provide a framework as support for medical graduates who wish to choose a medical specialty for their postgraduate training.

**Methods:**

In February 2020, physicians were invited via the State Chamber of Physicians’ monthly journal to electronically fill out the requirement tracking (R-Track) questionnaire. It contains 63 aspects assigned to six areas of competence: “Mental abilities”, “Sensory abilities”, “Psychomotor and multitasking abilities”, “Social interactive competences”, “Motivation”, and “Personality traits”. The expression of the different aspects was assessed on a 5-point Likert scale (1: “very low” to 5: “very high”). Sociodemographic data and information about the current workplace (hospital or practice) were also collected.

**Results:**

In total, 195 practicing physicians from 19 different specialities followed the invitation by the State Chamber of Physicians to participate in this survey. For almost all medical specialties, the competence area “Motivation” reached rank 1. “Psychomotor and multitasking abilities” received high ranks among specialties performing surgical activities, while “Social interactive competences” and “Personality traits” were highly rated by specialties with an intense level of patient-physician-interaction. “Mental abilities” were only rated highly by radiologists (rank 2) and physiologists (rank 3) while “Sensory abilities” were generally rated very low with the expression (rank 4) for anaesthesiology and ENT.

**Conclusions:**

In this pilot study, a first outline of competences profiles for 17 medical specialties were defined. The specific “Motivation” for a medical specialty seemed to play the greatest role for most specialties. This first specialty specific competence framework could provide a first insight into specific competences required by medical specialties and could serve medical graduate as a decision aid when looking for a medical specialty for their postgraduate training.

## Background

The medical specialties are characterized by a great diversity of their daily work. They differ not only in their work settings, needed skills, duties, responsibilities, amount of patient contact, type of patients, and working hours but also in prestige, status, social expectations and income [1, 2]. Taking this diversity into account, the decision making process and finally committing to a medical specialty for postgraduate medical education seems to be difficult for medical students [3, 4]. The individual choice of a medical speciality for postgraduate training is usually a decision for a lifelong career and, therefore, a very important step in the career path of a physician [5, 6]. To understand, how medical students decide to choose their specialty for postgraduate training, is also important for health service providers, government agencies, policy makers, and medical educators, because it determines the future of primary and specialized care in a society [4]. Regarding the shortage of physicians in some medical specialties, e.g. general practitioners or surgeons [7–9], the connection between postgraduate students’ specialty choice and physicians’ work power needs to be examined to ensure a long-term structure and composition of the health workforce [6, 10, 11].

The choice of a specialty for postgraduate training is a complex undertaking for medical graduates [12–14] and the career specialty decisions are influenced by many factors [15]. Prominent factors, which seem to have a high impact on specialty choice, include personality [1, 16], experiences in preclinical courses and clerkships [17, 18], role models [19, 20] or different gender preferences [21]. Other factors of relevance for the speciality selection are the amount of patient contact, geographic locations of practices, specialty-related income, and prestige including a growing interest in “controllable lifestyle” careers [15, 20–25]. From a more humorous perspective, certain characteristics have been attributed to medical specialities and have been collected in an algorithm for specialty decision making [26].

Finding a residency training position in a medical specialty works very differently according to a country's health and education system. While, e.g., in Germany graduates apply for a vacant position directly at a specific department of a hospital, The National Resident Matching Program (NRMP) of the United States of America provides a specific matching system of residency candidates with certain residency programs. The institutions reserve the right to reject a candidate if a more suitable applicant arrives [27, 28]. In order to make the right individual choice for a medical specialty, medical graduates need to reconcile their interests and values with their perception of the various specialist areas [6]. On the one hand, this concerns the graduates’ interests, needs and strengths, and on the other hand, the working conditions and opportunities a specific speciality contains [13, 29]. Because these are very different for the respective specialties, it is of central importance for the decision process to be familiar with the specific competences a certain specialty requires.

This pilot study aims to define competence profiles that reflect the specific requirements of different medical specialties. For this purpose, specialists from all medical specialties were invited to prioritize a set of competences for their specific daily work. These specialty specific competence profiles could provide multiple advantages: a guideline for medical graduates of what to expect in their favoured medical specialty and to evaluate their fit for a targeted specialty, an additional source of information for the design of postgraduate curricula and an orientation for personnel selection.

## Methods

### Study design, participants and instrument

To identify competence profiles of medical specialities, practicing physicians were invited to participate in this study via a link provided in Hamburger Ärzteblatt, a monthly journal in the German language, available in a print version for approximately 17,000 registered physicians in the state of Hamburg, Germany, and also available electronically for interested readers. The online survey of competences took place between February 1st and March 31st of 2020 on a voluntary basis. It contained the R-Track questionnaire, which was developed by Dr. Viktor Oubaid, psychologist at the German Aerospace Center. The questionnaire, originally designed to identify competence profiles of airline pilots, was adapted for health care professionals and other groups of professionals [30]. The design of R-Track is based on the Fleishman Job Analysis Survey (F-JAS) [31], which is a job analysis instrument of skills and abilities that are relevant for the accomplishment of certain occupational tasks or activities and can be used for all kind of professions [32].

The construction of the R-Track questionnaire is based on 6 areas of competence including a total of 63 questions: “Mental abilities” (14 questions, Cronbach’s alpha: .86), “Sensory abilities (9 questions, Cronbach’s alpha: .86), “Psychomotor and multitasking abilities” (2 questions, Cronbach’s alpha: .59), “Personality traits” (12 questions, Cronbach’s alpha: .78), “Motivation” (5 questions, Cronbach’s alpha: .62), and “Social interactive competences” (21 questions, Cronbach’s alpha: .87). All R-Track items are provided in Supplement 1. The competences are assessed on a 5-point Likert scale (1: very low importance, 2: low importance, 3: moderate importance, 4: high importance, 5: very high importance) with respect to their importance in the current workplace. Socio-demographic data including sex and workplace (hospital or practice) were also collected. This study was performed in accordance with the Declaration of Helsinki and the Ethics Committee of the Chamber of Physicians, Hamburg, confirmed the innocuousness of the study with consented, anonymized, and voluntary participation (PV3649). All participants provided informed written consent for participation in this study.

### Data processing

The R-Track questionnaire was presented in an online version. The assignments of items to these areas of competence were empirically proven by former data gained from medical requirement analysis [33]. Following Kleinmann et al. [32], only specialties with *n* ≥ 7 participants were included in the analysis. Mean scores and standard deviations were calculated for all subgroups using SPSS Statistics 25. ANOVA procedures were used for mean score comparison of subgroups and post hoc analyses were carried out with LSD test. Differences were considered significant for *p*-values < .05.

## Results

In total, 195 practicing physicians from 19 different medical specialties participated in the survey (anaesthesiology: *n* = 11, dermatology: *n* = 10, ENT: *n* = 13, forensic medicine: *n* = 2, general medicine: *n* = 11, gynaecology: *n* = 7, internal medicine: *n* = 20, neurology: n = 11, neurosurgery: *n* = 12, occupational medicine: *n* = 2, ophthalmology: *n* = 9, orthopaedics / trauma surgery: *n* = 11, paediatrics: *n* = 7, physiology: *n* = 12, psychiatry: *n* = 7, psychosomatic medicine: *n* = 14, radiology: n = 9, surgery: *n* = 14, and urology: *n* = 13). Their sociodemographic data are given in Table 1. Occupational medicine (*n* = 2) and forensic medicine (*n* = 2) were excluded from the data analysis due to the low number of participants.

**Table 1:** Sociodemographic data of participants

Medical specialty	Participants (n)	Sex (%)		Workplace (%)	
		Female	Male	Hospital	Practice
**Anaesthesiology**	11	45.5	54.5	90.9	9.1
**Dermatology**	10	50.0	50.0	100.0	0.0
**ENT**	13	30.8	69.2	92.3	7.7
**Forensic medicine**	2	50.0	50.0	100.0	0.0
**General Medicine**	11	90.9	9.1	45.5	54.5
**Gynaecology**	7	57.1	42.9	85.7	14.3
**Internal Medicine**	20	30.0	70.0	70.0	30.0
**Neurology**	11	81.8	18.2	100.0	0.0
**Neurosurgery**	12	25.0^a^	66.7^a^	91.7	8.3
**Occupational medicine**	2	50.0	50.0	50.0	50.0
**Ophthalmology**	9	55.6	44.4	66.7	33.3
**Orthopaedics / Trauma Surgery**	11	18.2	81.8	63.6	36.4
**Paediatrics**	7	28.6	71.4	71.4	28.6
**Physiology**	12	16.7	83.3	100.0	0.0
**Psychiatry**	7	57.1	42.9	57.1	42.9
**Psychosomatic Medicine**	14	64.3	35.7	100.0	0.0
**Radiology**	9	0.0	100.0	100.0	0.0
**Surgery**	14	50.0	50.0	100.0	0.0
**Urology**	13	23.1	76.9	69.2	30.8

^a^8.3% gave no gender information

The highest mean score (rank 1) was reached in the competence area “Motivation” by all participating medical specialties included in the data analysis except for ophthalmology (rank 1: “Psychomotor and multitasking abilities”) and psychiatry (rank 1: “Personality traits”) where “Motivation” reached rank 2 and 3, respectively (Table 2). The individual item with the highest score in the competence area “Motivation” was “Expertise”, the result of invested time and effort, for 14 specialities. “Personality traits” reached rank 2 by seven specialties and rank 3 by seven other specialties. The competence area “Psychomotor and multitasking abilities” received rank 2 by anaesthesiology, dermatology, ENT, neurosurgery, orthopaedics/trauma surgery, surgery, and urology. “Social interactive competences” reached rank 2 or 3, respectively, by ENT, general medicine, gynaecology, internal medicine, neurology, paediatrics, psychiatry, psychosomatic medicine, and radiology. The competence area “Mental abilities” was only ranked highly by radiologists and physiologists (rank 2 and 3, respectively). “Sensory abilities” reached ranks below 3 by all specialties with its highest rank being 4 only for anaesthesiology and ENT. ANOVA analyses revealed significant differences for medical specialties for all competencies except “Personality traits” with the largest differences in “Psychomotor and multitasking abilities” (F (16,174) = 10.45; *p* < .001), where specialties with a surgical focus had higher scores.

**Table 2:** Means and ranks of the six competence areas per specialty

	Competence area					
Medical specialty	Mental abilities MW ± SD (rank)	Sensory abilities MW ± SD (rank)	Psychomotor & multitasking abilities MW ± SD (rank)	Motivation MW ± SD (rank)	Social interactive competences MW ± SD (rank)	Personality traits MW ± SD (rank)
**Anaesthesiology** *n* = 11	3.63 ± 0.98 (6)	3.91 ± 0.90 (4)	4.09 ± 1.04 (2)	4.38 ± 0.43 (1)	3.79 ± 0.60 (5)	4.01 ± 0.44 (3)
**Dermatology** *n* = 10	3.83 ± 0.45 (5)	3.75 ± 0.42 (6)	4.30 ± 0.48 (2)	4.52 ± 0.53 (1)	3.91 ± 0.36 (4)	3.92 ± 0.51 (3)
**ENT** *n* = 13	3.79 ± 0.64 (6)	3.90 ± 0.67 (4)	4.42 ± 0.81 (2)	4.55 ± 0.34 (1)	4.06 ± 0.46 (3)	3.88 ± 0.65 (5)
**General Medicine** *n* = 11	3.32 ± 0.28 (4)	2.93 ± 0.55 (6)	3.05 ± 0.47 (5)	3.93 ± 0.45 (1)	3.66 ± 0.32 (3)	3.85 ± 0.49 (2)
**Gynaecology** *n* = 7	3.60 ± 0.84 (4)	3.38 ± 0.97 (6)	3.50 ± 0.91 (5)	4.06 ± 0.41 (1)	3.67 ± 0.36 (3)	3.75 ± 0.38 (2)
**Internal Medicine** *n* = 20	3.76 ± 0.59 (4)	3.43 ± 0.70 (5)	3.38 ± 0.72 (6)	4.36 ± 0.39 (1)	3.91 ± 0.41 (2)	3.89 ± 0.42 (3)
**Neurology** *n* = 11	3.57 ± 0.49 (4)	3.07 ± 0.47 (6)	3.55 ± 0.47 (5)	4.40 ± 0.45 (1)	3.61 ± 0.43 (3)	3.73 ± 0.34 (2)
**Neurosurgery** *n* = 12	3.86 ± 0.47 (6)	3.96 ± 0.55 (5)	4.58 ± 0.42 (2)	4.58 ± 0.39 (1)	3.98 ± 0.49 (4)	4.05 ± 0.46 (3)
**Ophthalmology** *n* = 9	3.55 ± 0.51 (5)	3.51 ± 0.38 (6)	4.39 ± 0.55 (1)	4.31 ± 0.46 (2)	3.69 ± 0.53 (4)	3.83 ± 0.40 (3)
**Orthopaedics / Trauma Surgery** *n* = 11	3.56 ± 0.44 (5)	3.28 ± 0.74 (6)	3.86 ± 0.67 (3)	4.55 ± 0.37 (1)	3.83 ± 0.39 (4)	4.00 ± 0.45 (2)
**Paediatrics** *n* = 7	3.37 ± 0.38 (4)	3.27 ± 0.57 (6)	3.36 ± 0.85 (5)	4.20 ± 0.58 (1)	3.79 ± 0.38 (3)	3.92 ± 0.54 (2)
**Physiology** *n* = 12	3.71 ± 0.38 (3)	2.85 ± 0.69 (6)	3.13 ± 1.19 (5)	4.33 ± 0.21 (1)	3.38 ± 0.54 (4)	3.73 ± 0.40 (2)
**Psychiatry** *n* = 7	3.62 ± 0.66 (4)	2.98 ± 0.88 (5)	2.57 ± 1.10 (6)	3.74 ± 0.46 (3)	3.74 ± 0.41 (2)	3.99 ± 0.35 (1)
**Psychosomatic Medicine** *n* = 14	3.50 ± 0.46 (4)	2.99 ± 0.55 (5)	2.11 ± 0.74 (6)	4.23 ± 0.51 (1)	4.10 ± 0.21 (3)	4.12 ± 0.40 (2)
**Radiology** *n* = 9	4.50 ± 0.53 (2)	4.06 ± 0.50 (5)	3.56 ± 0.77 (6)	4.73 ± 0.28 (1)	4.22 ± 0.59 (3)	4.17 ± 0.51 (4)
**Surgery** *n* = 14	3.65 ± 0.57 (5)	3.64 ± 0.46 (6)	4.43 ± 0.55 (2)	4.54 ± 0.44 (1)	3.85 ± 0.50 (4)	4.14 ± 0.47 (3)
**Urology** *n* = 13	3.62 ± 0.53 (5)	3.54 ± 0.73 (6)	4.12 ± 0.58 (2)	4.28 ± 0.44 (1)	3.73 ± 0.47 (4)	4.00 ± 0.52 (3)

## Discussion

Almost all medical specialties participating in our survey regarded “Motivation” as the most important competence area for their specialty with “Expertise” being the highest ranked individual item within this category for most specialties. This is an interesting finding, since the everyday work of medical experts is very different for specialties like, e.g. ophthalmology or psychosomatic medicine. However, this finding is in line with self-efficacy – a positive conviction of a person’s ability to successfully perform a certain action – being an essential aspect for motivation [34]. Enthusiasm for and commitment to a certain specialty have been shown to be among the most important aspects for junior doctors’ future specialty choice [35] and seem to be a continuing driving force for medical experts’ work in most specialties according to our findings. Nurturing medical students’ interest in surgery and providing motivating experiences in the surgical field during undergraduate training, for instance, increased medical graduates’ wish to choose surgery as specialty for their postgraduate education [36]. This could be a useful recruiting strategy for other specialties as well.

We also discovered interesting differences between the specialties with respect to the other five competence areas. As expected, “Psychomotor and multitasking abilities” were highly rated by physicians from all specialties that are involved in different kinds of surgical procedures. Most of these specialties require specific skills training, which begins during undergraduate medical education [37] and continues during postgraduate education where more specific psychomotor skills are needed depending on the specialty [38, 39]. Historically, surgeons have always been physicians who are involved with motorically challenging treatments and the divergence of surgery and medicine is still increasing [40]. Physicians from specialties where more narrative competences are required [41], e.g. internal medicine, general medicine or paediatrics, rated the competence areas “Social interactive competences” and “Personality traits” highly. For internists, a broad spectrum of interpersonal skills and communicative competences has been defined [42]. General medicine is associated with a high level of uncertainty [43], which requires a strong focus on the empathic interaction between physician and patient. For paediatricians, empathetic involvement with the patients’ parents is of great importance because of the often heterogeneous and non-specific symptoms of the patients [44]. For physicians working in psychiatry and psychosomatic medicine, empathetic understanding plays an important role for their therapeutic relationships with the patients [45]. “Emotional stability” and “Openness to other people/cultures” were rated as the most important aspects in the competence areas “Social interactive competences” and “Personality traits” by most of these specialties.

The competence area “Mental abilities” was only rated highly by radiologists and physiologists. This could be due to the fact that radiologists spend a lot of time on the reconstruction of two- or three-dimensional imaging [46, 47] and physiologists’ field of work focuses on abstract thinking [48]. Medical graduates who wish to choose either specialty for their postgraduate training need to be aware that – besides motivation – their specific focus and interest should lie on mental abilities required as specifically prominent area of competence for radiology and physiology. “Sensory abilities” were rated relatively low by most specialties but received the highest scores by anaesthesiology and ENT. Anaesthesiologists need good auditory discrimination and selective attention when monitoring anaesthetized patients [49]. For ENT-specialists, visual imagination and range of field vision are required to work in complex and narrow three dimensional spaces. These specialty-specific skills can be trained with mannequins [50] or in virtual reality simulations [51].

The identified competence profiles could provide a guideline for medical graduates of what to expect in their favoured medical specialty. Based on our survey, three main recommendations can be given to medical graduates who wish to choose a specialty for their postgraduate training. The most important aspect is to identify their individual intrinsic motivation for a specific specialty. Hence, medical graduates should explore their curiosity, the main intrinsic motivation of learning, with respect to a specialty of their choice, and their self-efficacy, the primary factor of learning motivation, which have been shown to influence each other [52]. A second decision could be whether graduates are interested in a specialty that specifically requires rather psychomotor or psychosocial skills. Psychomotor skills are prominently needed by all specialties who perform surgical procedures. This supposedly still reflects the development of the surgical specialties from the handicraft of barbers to surgery as a science [53] with a main focus lying on manual skills. Psychosocial skills are highly required by the different specialties belonging to the so called “talking medicine” [54]. Once this decision is made, the third aspect of specialty selection should be on specific requirements like mental or sensory abilities.

Our study has several limitations. As a pilot study, it was limited to one country and the invitation to participate was provided via an analogue physicians’ journal distributed in one area of Germany and available in the internet. Therefore, we are not able to provide a response rate. Furthermore, part of the survey period coincided with the shutdown due to the COVID-19 pandemic which might additionally have hampered the response to the questionnaire. However, we received enough responses to analyse 17 of 19 participating specialties. Additionally, the Cronbach’s alphas for four areas of competence are good except for “Psychomotor and multitasking abilities” and “Motivation”, which include only two and five items, respectively. Another limitation is that we did not collect the age of the participants, which could play a role, since some medical specialties changed over time and, e.g. radiologists do very different things than a generation ago. This pilot study enabled us to provide a first insight into competence profiles of different specialties. Studies with a larger number of participants within each specialty or even subspecialty and including the age of the participants are needed to consolidate our pilot findings. These first insights into competence profiles might already serve as an initial decision aid for medical graduates what to expect form a certain specialty for postgraduate training. Investigating medical students’ competence profiles and matching them with the profile of their desired specialty for postgraduate education would be an interesting next step.

## Conclusions

Different competence profiles for medical specialities were discovered in this pilot study. The competence area “Motivation” reached the highest rank in almost all specialties. Additionally, many specialties either ranked “Psychomotor and multitasking abilities” or “Social interactive competences” and “Personality traits” highly. These findings could provide a first insight into specific competences required by medical specialties and support medical graduates in making their choices for a specialty for postgraduate training.

## Data Availability

All data and materials are available from the manuscript and from the corresponding author upon request.

## Abbreviations

ANOVA: Analysis of variance
COVID-19: Corona Virus Disease 2019
ENT: ears, nose, throat
F-JAS: Fleishman job analysis survey
NRMP: National resident matching program
LSD: Least significant difference test
R-Track: requirement-tracking
SPSS: Statistical package for the social sciences
